# Autoimmune GFAP astrocytopathy associated with sintilimab immunotherapy in a patient with esophageal squamous cell carcinoma: a case report and literature review

**DOI:** 10.3389/fonc.2026.1866471

**Published:** 2026-07-24

**Authors:** Yajie Fan, Yaxuan Yao, Xutao Guan, Shansi Zou, Menghua Li, Qilong Gao, Qingyun Hao, Xinmeng Zhang, Tao Wang, Shiling Sun

**Affiliations:** 1 Department of Haemato-Oncology, The First Affiliated Hospital of Henan University of Traditional Chinese Medicine, Zhengzhou, Henan, China; 2 The First Clinical Medical College of Henan University of Traditional Chinese Medicine, Zhengzhou, Henan, China; 3 Henan University of Traditional Chinese Medicine, Zhengzhou, Henan, China; 4 Collaborative Innovation Center of Prevention and Treatment of Major Diseases by Chinese and Western Medicine, Zhengzhou, Henan, China

**Keywords:** autoimmune GFAP astrocytopathy, case report, esophageal squamous cell carcinoma, immune-related adverse events, PD-1 inhibitor, sintilimab

## Abstract

Esophageal squamous cell carcinoma (ESCC) is a highly lethal malignant tumor. Immune checkpoint inhibitors (ICIs), represented by sintilimab, have become an important treatment option for advanced ESCC, but they may induce immune-related adverse events (irAEs) involving multiple systems, among which neurological irAEs, though rare, can be severe. Autoimmune glial fibrillary acidic protein (GFAP) Astrocytopathy is a novel autoimmune disorder of the central nervous system (CNS). Its occurrence in ESCC patients receiving sintilimab immunotherapy has not been previously documented in detail. This report presents a rare case of Autoimmune GFAP Astrocytopathy in a 55-year-old male patient with advanced ESCC following sintilimab (200 mg) combined with paclitaxel and cisplatin chemotherapy. Additionally, a systematic literature review identified 17 cases of immune-associated encephalitis induced by PD-1 inhibitors, whose clinical features were similar to those in this case. This case provides detailed clinical, laboratory and imaging data of sintilimab-related autoimmune GFAP astrocytopathy in a patient with advanced esophageal squamous cell carcinoma, supplementing real-world clinical evidence of CNS immune-associated adverse events induced by sintilimab. We explore the potential pathogenesis, diagnostic criteria, and treatment strategies for this condition. The patient achieved complete remission after early high-dose glucocorticoid pulse therapy combined with intravenous immunoglobulin (IVIG), with sustained neurological remission observed during a 3-month outpatient observation window (partial completion of the planned 6-month steroid tapering regimen).This study emphasizes the need for clinical vigilance regarding Autoimmune GFAP Astrocytopathy in ESCC patients presenting with neurological manifestations during PD-1/PD-L1 inhibitor therapy, and highlights that early detection, prompt discontinuation of ICI, and aggressive immunosuppressive therapy are critical for improving patient outcomes. These findings provide valuable clinical insights for the early diagnosis and individualized management of CNS irAEs associated with PD-1 inhibitor therapy in ESCC patients. Notably, only a temporal association between sintilimab therapy and disease onset can be confirmed; direct causal evidence remains unproven.

## 1 Introduction

Esophageal squamous cell carcinoma (ESCC) is one of the most lethal malignancies globally, with high incidence and mortality rates, particularly in Eastern Asia (1).For advanced or metastatic ESCC, conventional treatments such as surgery, radiotherapy, and chemotherapy have limited efficacy and are often accompanied by severe adverse reactions, resulting in an unsatisfactory prognosis (2). In recent years, immune checkpoint inhibitors (ICIs), including anti-PD-1 and anti-PD-L1 monoclonal antibodies, have revolutionized the treatment landscape of advanced ESCC by reactivating the host’s anti-tumor immune response, thereby significantly improving objective response rates and overall survival in selected patients (3).

Sintilimab is a fully humanized anti-PD-1 monoclonal antibody that specifically binds to PD-1, blocks the PD-1/PD-L1 signaling pathway, and enhances the cytotoxic activity of T lymphocytes against tumor cells (4).It has been approved for the first-line treatment of advanced ESCC in China and other countries, with a favorable safety profile. Despite this, the activation of the immune system by ICIs can also trigger a series of immune-related adverse events (irAEs) involving multiple organs and systems (5–7), not only including the skin, gastrointestinal tract, andendocrine system, but also including exacerbation or production of autoimmune neurological disorders, such as autoimmune encephalitis (8), myelitis (9), Guillain-Barré syndrome (10), and neuromuscular disorders (11). Among neurological irAEs, symptoms of these diseases are relatively rare but can be severe and even fatal if not promptly recognized and treated.

Autoimmune glial fibrillary acidic protein (GFAP) astrocytopathy is a novel CNS autoimmune disease first described by Fang et al. in 2016, characterized by the presence of anti-GFAP IgG antibodies (predominantly targeting the GFAPα isoform) in the CSF and serum, as well as immune-associated inflammation of astrocytes (12). The disease exhibits a heterogeneous clinical spectrum, most commonly presenting as meningoencephalitis (with or without myelitis), and may also manifest as headache, visual disturbances, extrapyramidal symptoms, brainstem syndromes, or psychiatric manifestations. The exact pathogenic mechanisms of GFAP astrocytopathy remain unclear; however, it is hypothesized that molecular mimicry associated with infections, tumors, or immunotherapies may lead to the production of anti-GFAP antibodies and the activation of GFAP-specific CD8 T cells, resulting in astrocyte damage and neuroinflammation (13). Although GFAP astrocytopathy has been reported in association with various tumors and active viral infections, mNGS only detected low-load latent EBV fragments in our patient’s CSF without clinical or laboratory evidence of acute viral encephalitis. Neurological manifestations arose shortly after sintilimab infusion; definitive causal evidence linking sintilimab to autoimmune GFAP astrocytopathy cannot be established, and only a temporal correlation can be confirmed.

Herein, we report a rare case of autoimmune GFAP astrocytopathy in a patient with advanced ESCC following sintilimab treatment, and conducted a comprehensive literature review to discuss the clinical characteristics, diagnosis, treatment, and prognosis of this disease. This case aims to highlight the need for heightened clinical suspicion of autoimmune GFAP astrocytopathy in patients developing neurological manifestations during PD-1/PD-L1 blockade, raise clinicians’ awareness of this disease and emphasize the importance of early detection and intervention, and provide a reference for the management of similar cases in clinical practice.

## 2 Case presentation

A 55-year-old male presented to our department with a 20-day history of esophageal malignant tumor accompanied by mental confusion for 3 days. Neurological examination revealed spontaneous involuntary limb movements; muscle strength was approximately grade 4+ in all extremities, with normal muscle tone and symmetric tendon reflexes. Bilateral pathological reflexes (including Babinski sign, Chaddock sign, Oppenheim sign and Gordon sign) were all negative. No neck rigidity or meningeal irritation signs were observed, and the patient was unable to cooperate with complete higher cognitive function assessment.

The patient had no other underlying medical conditions. He was diagnosed with esophageal squamous cell carcinoma (ESCC,T3N0M0,stage IIB) on January 31,2026. The patient initiated the first cycle of chemotherapy combining paclitaxel and cisplatin on February 3, 2026. Two days later (February 5, 2026), a single intravenous infusion of sintilimab (200 mg) was administered to complete the first cycle of combined chemotherapy and immune checkpoint inhibitor therapy, during which no related adverse events were observed. On February 21, 2026 (16 days following single-dose sintilimab infusion, representing a clear temporal interval between immunotherapy exposure and symptom onset), initial neurological symptoms including drowsiness, confusion, fever, dizziness, nausea, and hiccups occurred. On February 22,2026, sintilimab and subsequent planned chemoradiotherapy were permanently discontinued due to the identification of immune-associated neurological adverse events. Initiation of first-line immunosuppressive rescue therapy: methylprednisolone pulse 1000 mg IV daily ×5 days plus concurrent IVIG (total cumulative dose 2 g/kg divided over 5 consecutive days) on February 23,2026. Subsequent follow-up evaluations demonstrated gradual resolution of confusion and involuntary limb movements within 7 days (02 March 2026) post-immunosuppressive therapy. During the fourth-week follow-up (21 March 2026), repeat lumbar puncture and cranial MRI performed; CSF GFAP antibody turned negative, CSF biochemistry normalized, and periventricular white matter hyperintensities completely resolved. Oral prednisone tapering regimen was initiated at 60 mg daily, with a 5 mg reduction every two weeks; planned full tapering duration was 6 months. For the 3-month clinical observation window (ending June 10, 2026, 74 days post discharge on March 28, 2026), the patient only completed the first half of the full tapering protocol and temporarily suspended oral prednisone maintenance under multidisciplinary neurological and oncologic joint assessment due to stable complete neurological remission and tolerable esophageal tumor status. Full neurological recovery without residual deficits was achieved on discharge date (28 March 2026). Of note, all follow-up data presented herein are limited to a 3-month observation window ending on June 10, 2026. During this 3-month outpatient follow-up period, no recurrent neurological symptoms were observed in the patient, and maintenance oral prednisone was temporarily suspended as of June 10, 2026.Given the permanent discontinuation of sintilimab due to severe immune-associated neurological adverse events, the multidisciplinary oncology team adjusted the subsequent anti-tumor strategy for esophageal squamous cell carcinoma. The patient received single-agent paclitaxel chemotherapy every 3 weeks as second-line systemic treatment, without additional immunotherapy or thoracic radiotherapy during the 3-month follow-up window. Serial chest and abdominal CT scans every 4 weeks demonstrated stable esophageal primary lesions without distant metastasis or local progression, and the patient maintained good tolerance to single-agent paclitaxel without new grade ≥2 adverse reactions.

Laboratory findings revealed a prior resolved hepatitis B virus (Laboratory findings revealed a prior hepatiHBV) infection: serological tests showed positive hepatitis B surface antibody, negative hepatitis B surface antigen, positive hepatitis B core antibody, with undetectable peripheral blood HBV-DNA at admission, confirming complete clinical recovery without chronic active hepatitis B. Abnormal liver function parameters included elevated alanine aminotransferase (132.5 U/L, normal range 0-50), elevated glutamyl transpeptidase (117.7 U/L, normal range 0-60), decreased total protein (57.6 g/L, normal range 65-85), and decreased albumin (35.7 g/L, normal range 40-55). Renal function abnormalities included decreased creatinine (49.5 μmol/L, normal range 59-97) and decreased uric acid (153.0 μmol/L, normal range 202-416). Coagulation function showed elevated fibrinogen (4.44 g/L, normal range 2-4). Tumor markers demonstrated increased ferritin (503.0 ng/ml, normal range 23.9-336.2), while AFP, CEA, CA199, CA125, CA153, and CA724 remained within normal ranges.

**Figure 1:**
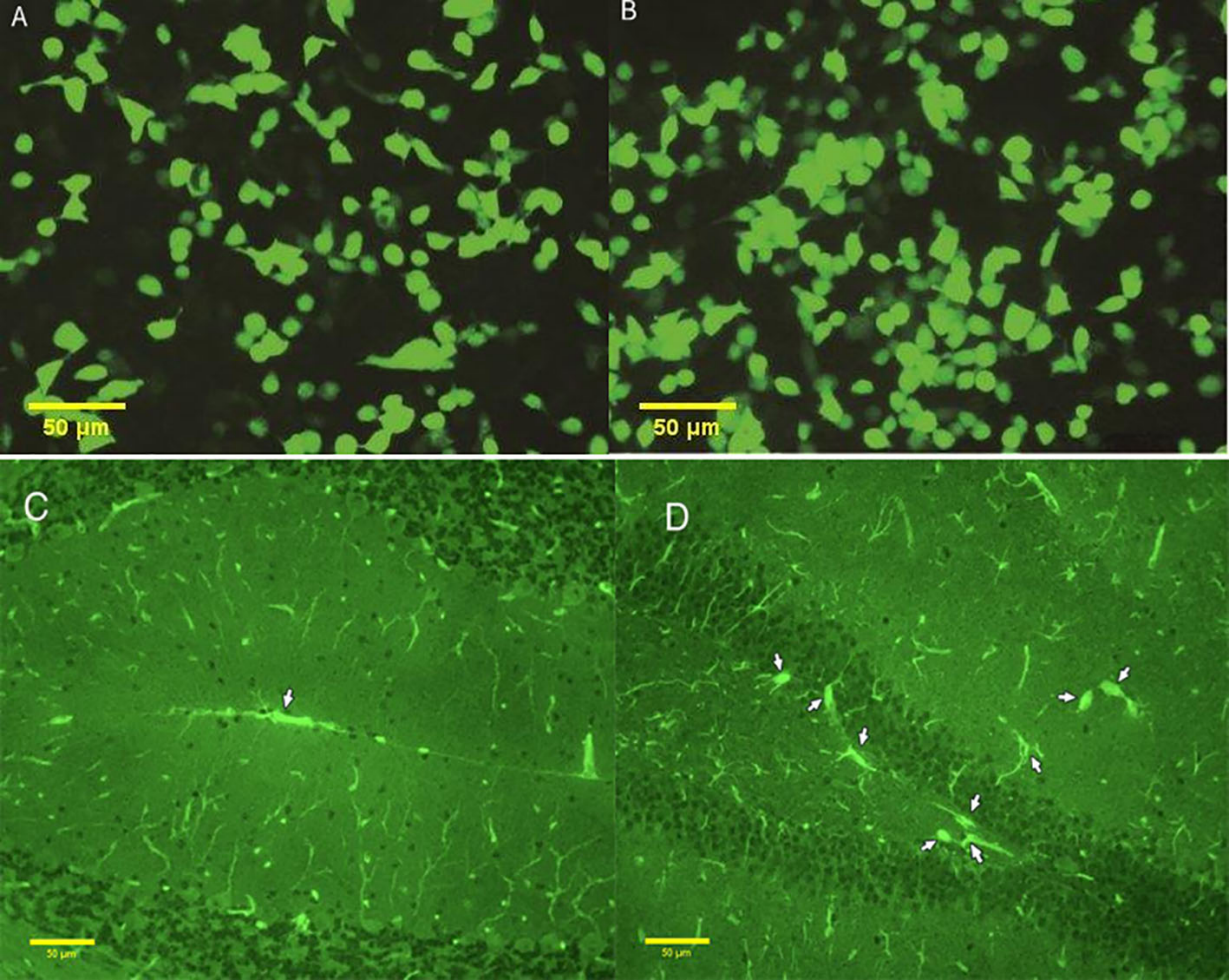
Cell-based and tissue-based methods were employed to detect serum, CSF, and GFAP antibodies in patients. Indirect immunofluorescence staining revealed weak positivity for GFAP antibodies in CSF, with a titer of 1:3.2 **(A)**, while the corresponding isotype control was negative **(B)**. Qualitative tissue-based assay (TBA) detection on brain tissue sections showed weak positive GFAP antibody staining reactions, with characteristic radial fluorescent signals observed in the molecular layers of the cerebellum **(C)** and hippocampus **(D)**.Original magnification: ×200.

CSF analysis demonstrated intracranial pressure measured by lumbar puncture at 270 mmH_2_O, with colorless and transparent cerebrospinal fluid (CSF). Paine’s test (a qualitative bedside screening test for CSF pleocytosis and elevated protein using glacial acetic acid precipitation) yielded negative results. The CSF routine leukocyte count was 2×10^6^/L, while CSF total protein was elevated to 112 mg% (reference range:15–45 mg%). CSF bacterial culture remained sterile for 48 hours. Centrifuged smear and thin-layer cytology (TCT) demonstrated moderate lymphocytes and neutrophils upon light microscopy, along with a small number of histiocytes and monocytes, but no definitive malignant tumor cells were detected. This apparent discrepancy between low routine cell count and sparse moderate inflammatory cells on cytology slides is fully explained by pre-analytical technical differences: routine uncentrifuged CSF white blood cell count remained within normal limits (no pathological pleocytosis consistent with infectious encephalitis); sparse moderate lymphocytes and neutrophils were only observed after high-speed centrifugation and concentration for thin-layer cytology (TCT). This concentration artifact does not represent true elevated intrathecal inflammatory cells, and no pathologically significant leukocytosis was detected in native CSF samples. Expanded autoantibody screening was performed via cell-based assay (CBA) and tissue-based assay (TBA) for GFAP antibody detection. The laboratory diagnostic cutoff for positive GFAP antibody by CBA in cerebrospinal fluid (CSF) was defined as titer ≥1:3.2; serum GFAP antibody testing was negative at 1:1.0 (below the assay cutoff). The CSF sample was retested twice with identical CBA and TBA protocols, and both repeated tests confirmed weak GFAP antibody positivity at a consistent titer of 1:3.2 (**Figures 1A, B**). Qualitative TBA staining on fixed brain tissue slides also yielded weak positive signals with typical radial immunofluorescence patterns in the cerebellar and hippocampal molecular layers (**Figures 1C, D**). Parallel serum and CSF panels for 14 paraneoplastic neuronal antibodies (anti-Hu, anti-Ri, anti-Yo, etc.) were uniformly negative. Metagenomic next-generation sequencing (mNGS MetaCAP platform) of CSF identified only trace, low-load latent EBV nucleic acid fragments without any genomic markers of active EBV lytic replication; no other pathogenic bacteria, fungi, or neurotropic viruses were detected in CSF. The trace low-load latent EBV nucleic acid fragments identified by CSF mNGS only represent quiescent endogenous latent viral reactivation secondary to PD-1 immune tolerance breakdown, without any molecular markers of EBV lytic replication, elevated EBV viral load, or clinical manifestations of acute EBV encephalitis. This incidental latent viral signal cannot independently drive astrocytic inflammatory injury, so we definitively excluded primary active viral meningoencephalitis as the core etiology and withheld empirical antiviral medication throughout hospitalization. No oligoclonal bands were detected in CSF or serum. Although neurological symptoms developed shortly after sintilimab administration, only temporal correlation can be confirmed, and definitive causal evidence linking sintilimab to autoimmune GFAP astrocytopathy is absent.

This study conducted a systematic differential diagnosis by integrating the patient’s clinical symptoms, cranial imaging data, cerebrospinal fluid and serum immunological parameters, as well as genetic sequencing results, systematically ruling out various central nervous system disorders with similar clinical manifestations. For infectious meningoencephalitis, mNGS of cerebrospinal fluid detected only low-copy latent EBV fragments without molecular markers of acute active viral lytic infection; bacterial cultures yielded no pathogenic organisms. These low-abundance latent EBV nucleic acid fragments do not confirm active viral infection and are not sufficient to diagnose EBV encephalitis. Although sparse moderate inflammatory cells were only visible after high-speed centrifugation and slide concentration for CSF cytology, routine uncentrifuged native CSF cell count remained within normal limits without pathological pleocytosis, cranial MRI lacked characteristic infectious parenchymal or meningeal lesions, and no clinical manifestations of acute viral encephalitis (persistent high fever, severe meningeal irritation, EBV lymphoproliferative lesions) were observed throughout hospitalization. No empirical antiviral therapy was administered during hospitalization; we therefore excluded acute bacterial or active viral meningoencephalitis as the primary etiology without relying on antiviral treatment response. No diffuse slow waves, epileptiform discharges, or focal encephalopathy changes were detected during electroencephalography (EEG) examination. The expanded panel of autoimmune encephalitis antibody tests—including comprehensive serum and cerebrospinal fluid assays for NMDA receptors, LGI1, CASPR2, AMPA1/2 receptors, GABA-B receptors, and mGluR5—all returned negative results. Metabolic and toxicological screening parameters (serum ammonia, lactate levels, thyroid function, heavy metal toxicity profiles, as well as vitamin B12 and folic acid concentrations) fell within normal reference ranges, ruling out the possibility of metabolic or toxic encephalopathy. The extended infectious disease testing—including cerebrospinal fluid cryptococcal antigen analysis, tuberculosis PCR, syphilis serology, and serological tests for herpes simplex virus, varicella-zoster virus, and cytomegalovirus—all yielded negative results, further excluding other central nervous system infections. For paraneoplastic encephalitis, serum and cerebrospinal fluid tests for paraneoplastic neuronal antibodies (anti-Hu, anti-Ri, anti-Yo, etc.) were all negative; chest and cranial imaging screening identified no occult tumor metastatic lesions, supporting no diagnosis of paraneoplastic autoimmune central nervous system disease. For leptomeningeal metastasis, cerebrospinal fluid cytology failed to detect tumor cells; cranial magnetic resonance imaging (MRI) showed no typical imaging findings such as abnormal enhancement of the soft meninges, meningeal nodules, or communicating hydrocephalus; and serum levels of broad-spectrum tumor markers remained within normal ranges, confirming the exclusion of leptomeningeal metastasis. For other immune checkpoint inhibitor (ICI)-related neurological adverse reactions, the diagnosis can be differentiated from ICI-associated myelitis, Guillain-Barré syndrome, myasthenia gravis, and non-specific autoimmune encephalitis with negative GFAP antibodies by considering the patient’s clinical characteristics, positive cerebrospinal fluid glial fibrillary acidic protein (GFAP) antibody results, and characteristic immunofluorescence expression patterns. Based on the patient’s distinctive clinical phenotype, weak CSF GFAP antibody positivity confirmed by duplicate CBA/TBA testing, negative comprehensive auxiliary examinations and full exclusion of alternative etiologies, the final clinical diagnosis was probable sintilimab-associated autoimmune GFAP astrocytopathy.

Cranial MRI showed no definite DWI hyperintense lesions within the brain parenchyma; faint white matter hyperintensities were observed in bilateral periventricular white matter and corona radiata (Fazekas grade 1), vacuolar sella turcica, bilateral ethmoid sinus and maxillary sinusitis, and a small cyst in the left maxillary sinus. These findings ruled out causes such as brain metastases or parenchymal brain lesions. Chest CT revealed no abnormal structures. Combined with all differential testing results, the final clinical diagnosis was probable sintilimab-associated autoimmune GFAP astrocytopathy.

The patient received a standardized first-line immunosuppressive regimen immediately after diagnosis: methylprednisolone pulse therapy at 1000 mg intravenous daily for 5 consecutive days, followed by oral prednisone tapering (starting dose 60 mg/d, gradually reduced by 5 mg every 2 weeks over 3 months). Concurrent intravenous immunoglobulin (IVIG) was administered at a total cumulative dose of 2 g/kg, split into 5 daily infusions. At 4-week follow-up, repeated lumbar puncture showed CSF leukocyte count returned to normal (0 ×10^6^/L), CSF protein decreased to 38 mg%, and repeat CBA GFAP antibody testing turned negative (titer <1:3.2). Follow-up cranial MRI revealed complete resolution of faint periventricular white matter hyperintensities. The patient maintained full neurological remission without symptom recurrence for 3 months after discharge, with no requirement for maintenance immunosuppressive therapy.

**Figure 2:**
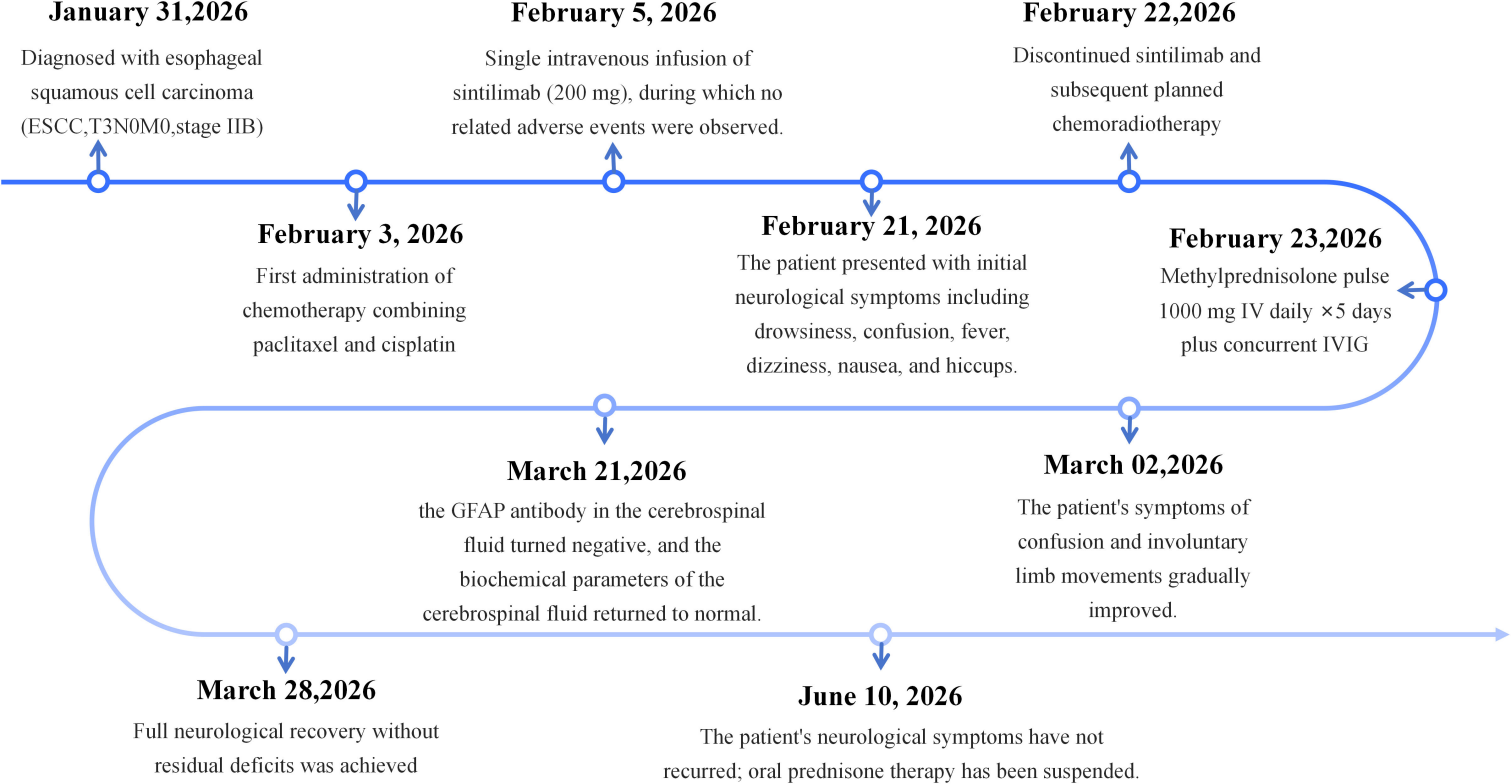
Clinical timeline of disease onset, treatment and follow-up of the patient.

To intuitively clarify the temporal sequence of sintilimab exposure, neurological symptom occurrence, discontinuation of PD-1 inhibitor, initiation of rescue immunosuppressive therapy, seroclearance of CSF GFAP antibody, resolution of cranial imaging lesions and complete neurological recovery, we constructed a detailed clinical timeline as shown in **Figure 2**.

## 3 Literature review

A systematic literature retrieval was performed before June 15, 2026, covering four mainstream biomedical electronic databases, namely PubMed, Embase, Web of Science and China National Knowledge Infrastructure (CNKI).The retrieval strategy was formulated by combining subject terms and free words, and the detailed search formula was listed as follows: (sintilimab OR pembrolizumab OR nivolumab OR PD-1 inhibitor) AND (GFAP astrocytopathy OR autoimmune encephalitis OR neurological immune-related adverse events OR meningoencephalomyelitis).

The inclusion criteria were set as follows: (1) original case reports or case series focusing on autoimmune encephalitis associated with programmed death-1 (PD-1) inhibitor therapy; (2) full-text articles with complete clinical manifestation data, cerebrospinal fluid (CSF) test results, neuroimaging findings and follow-up prognosis information; (3) study subjects were adult tumor patients and no pediatric patients were included. Correspondingly, the exclusion criteria included review articles, animal experimental studies, conference abstracts without complete full texts, repeatedly published literature, and clinical cases treated with single programmed death ligand-1 (PD-L1) inhibitor rather than PD-1 inhibitor monotherapy or combined therapy.

Key outcome indicators were extracted independently, including treatment response rate, disease relapse rate and all-cause mortality. All extracted data were manually cross-checked and verified against the original full-text literature by two researchers to eliminate subjective deviation and ensure the accuracy and reliability of pooled data.

Through systematic literature review, we identified 17 cases of immune-associated encephalitis following PD-1 inhibitor therapy (14–27). All literature retrieval for quantitative pooled analysis was performed with a cutoff date of June 15, 2026, which covers all articles published in 2026 included in **Table 1**. The median age at onset was 61 years (range: 40–83 years) and there was a male predominance (10/17,58.8%). The main types of malignant tumors included lung cancer (8/17,47.1%) and melanoma (6/17,35.3%). Pembrolizumab(4/17, 23.5%)and Nivolumab(8/17, 47.1%)were the most commonly used PD-1 inhibitors in clinical practice. CSF analysis revealed median pleocytosis of 49.3 cells/L(range 2-1502) and elevated protein levels (median 0.73 g/L, range 0.32-4.79). Seven patients tested positive for autoantibodies: one serum anti-Sox1 antibodies positive case (24), one serum Anti-AQP4 antibodies positive case (17), four previously reported PD-1 inhibitor-related autoimmune GFAP astrocytopathy cases, including one sintilimab-related case from Ref (14) and three additional cases from Refs (15–17),and one CSF anti-Sox1 antibodies positive case (24).Most cranial MRI scans showed no abnormal imaging findings, with 35.3% (6/17) demonstrating enhanced signals in brain parenchyma. All patients received ICI withdrawal combined with immunosuppressive therapy: high-dose glucocorticoid monotherapy (6/17,35.3%) or glucocorticoid combined with other immunotherapies (11/17,64.7%). Although most patients responded well to glucocorticoid therapy (14/17,82.3%), disease relapse occurred in 35.3% (6/17) of patients, necessitating further escalation of immunotherapy. The overall sustained clinical remission rate after standardized immunosuppressive intervention was 64.7% (11/17); transient clinical improvement (partial symptom relief within 2 weeks) was observed in 12/17 patients as summarized in **Table 1**. Among the 17 pooled PD-1 inhibitor-associated autoimmune encephalitis cases summarized in **Table 1**, only four patients were confirmed with CSF anti-GFAP antibody positivity, corresponding to rare autoimmune GFAP astrocytopathy associated with PD-1 inhibitor therapy, which is the core disease focus of the present study. To eliminate clinical heterogeneity and facilitate targeted comparison of GFAP-positive cases, we extracted these four documented GFAP astrocytopathy cases and listed them separately in **Table 2** for subgroup analysis.

**Table 1:** Summary of published cases with PD-1 inhibitor-related immune-associated encephalitis reported prior to June 15, 2026.

Items	All patients
Demographics	
Age at encephalitis onset, median (range), years	61 (40-83)
Male	10
Female	7
Malignancy	
Lung cancer	8(47.1)
Melanoma	6(35.3)
Others	3(17.6)
ICI treatment	
Pembrolizumab	4(23.5)
Nivolumab	8(47.1)
Nivolumab and ipilimumab	4(23.5)
Sintilimab	1(5.9)
Clinical manifestation	
Encephalitis	12(70.6)
Myelitis	4(23.5)
Encephalomyelitis	1(5.9)
CSF findings	
Cells (cells/μL), median (range) (n = 12)	49.3 (2-1,502)
Proteins (g/L), median (range) (n = 13)	0.73 (0.32-4.79)
Restricted OCB (n = 9)	3 (33.3)
Serum autoantibodies	
Anti-SOX1 antibody	1/17
Anti-AQP4 antibodies	1/5
CSF autoantibodies	
Anti-GFAP antibodies	4/9
MRI involvement	
Parenchymal enhancement on MRI	6/17(35.3)
Treatment	
Glucocorticoid monotherapy	6(35.3)
Glucocorticoids and other immune therapies	11(64.7)
Responsiveness to steroids	14(82.3)
Relapse	6(35.3)
mRS at onset, median (range)	4(1-5)
mRS at last follow-up, median (range)	3(0-6)
Outcome at last follow-up	
Clinical improvement	12(70.6)
No improvement	3(17.6)
Death	2(11.8)
Follow-up from encephalitis onset (months), median (range)	7(2-26)

*Definition distinction: Steroid responsiveness = symptom relief within 14 days of steroid initiation; Clinical improvement = any transient symptom alleviation during follow-up; Clinical response = sustained stable remission at final follow-up.

**Table 2:** Summary of reported autoimmune GFAP astrocytopathy cases associated with PD-1 inhibitor therapy with confirmed anti-GFAP antibody positivity.

Age (years)	40	47	70	55
Sex	Male	Male	Male	Male
Underlying malignancy	HCC	Metastatic melanoma	NSCLC	NSCLC
PD-1 inhibitor	sintilimab	Nivolumab	Nivolumab	Pembrolizumab
Clinical syndrome	Myelitis	Encephalitis	Encephalitis	Encephalitis
Autoantibody status	CSF GFAP antibody positive	CSF GFAP antibody positive	CSF GFAP + NMDA receptor antibody positive	Serum + CSF GFAP antibody positive
Baseline mRS score	2	3	4	3
Treatment regimen	Prednisone 40mg IV daily for 3 days	Methylprednisolone 1g IV daily for 5 days + 5 plasma exchange sessions	Methylprednisolone 1g IV daily for 5 days	Methylprednisolone 1g IV daily for 5 days
Clinical improvement	Yes	Yes	Yes	No
Relapse	No	No	Yes	No
Survival outcome	Alive	Alive	Alive	Alive
Follow-up duration (months)	9	4	6	24
References	(14)	(15)	(16)	(17)

*All clinical data of the four GFAP-positive cases were rechecked and extracted strictly according to the original full text of References 14–17 to ensure data accuracy.

As shown in **Table 2**, all four literature-reported GFAP astrocytopathy patients were male, with onset ages ranging from 40 to 70 years old. The underlying malignancies included hepatocellular carcinoma, metastatic melanoma and non-small cell lung cancer, covering three types of tumors without esophageal squamous cell carcinoma (ESCC). Two types of PD-1 agents were involved: sintilimab (1 case for HCC) and anti-PD-1 monotherapy of nivolumab/pembrolizumab (3 cases for melanoma/NSCLC). Clinical phenotypes were divided into isolated myelitis and encephalitis; one case co-expressed CSF anti-NMDA receptor antibody, showing overlapping autoimmune antibody spectrum. For treatment strategies, all patients received systemic glucocorticoid therapy, while plasma exchange was added for one severe melanoma case. Most patients achieved neurological improvement, with only one NSCLC patient presenting persistent non-response and one nivolumab-treated encephalitis case suffering disease relapse during follow-up. All four patients survived long-term follow-up ranging from 4 to 24 months.

Notably, **Table 2** does not contain ESCC-related cases of GFAP astrocytopathy associated with sintilimab exposure, which represents the novelty of our current research. Compared with the four literature cases in **Table 2**, the ESCC patient in this study exhibited distinct tumor background, combined paclitaxel/cisplatin immunochemotherapy regimen and typical meningoencephalitis manifestations, receiving combined methylprednisolone pulse plus IVIG therapy and achieving complete 3-month sustained remission without relapse.

## 4 Discussion

Multiple cases of autoimmune GFAP astrocytopathy associated with sintilimab exposure have been documented to date, and the present case further supplements clinical evidence of severe CNS immune-related complications in patients with esophageal squamous cell carcinoma receiving PD-1 inhibitor therapy.

Autoimmune GFAP Astrocytopathy is a rare CNS irAE associated with ICIs, and its clinical characteristics are heterogeneous (5, 7). According to previous literature reports, the median age of onset of Autoimmune GFAP Astrocytopathy is 40–60 years, with no obvious gender difference. The most common clinical manifestations are meningoencephalitis, headache, fever, and altered mental status, which are consistent with the symptoms of the patient in this case.

In terms of diagnosis, This case fulfilled clinical criteria for probable autoimmune GFAP astrocytopathy secondary to sintilimab immunotherapy, with weak CSF GFAP antibody positivity confirmed by duplicate CBA and TBA testing, accompanied by typical clinical manifestations such as fever, dizziness, and confusion. Imaging studies excluded brain metastases and parenchymal brain lesions, meeting the diagnostic criteria for Autoimmune GFAP Astrocytopathy.

The exact pathogenic mechanisms of autoimmune GFAP astrocytopathy associated with PD-1 inhibitor therapy remain unclear, but several hypotheses have been proposed. First, the molecular mimicry hypothesis: PD-1 inhibitors activate the immune system, leading to the proliferation and activation of T lymphocytes. Tumor cells or viral antigens may share similar epitopes with GFAP, leading to cross-reactivity of activated T lymphocytes and antibodies with astrocytes, resulting in astrocyte damage and neuroinflammation (28). Second, the immune dysregulation hypothesis: PD-1/PD-L1 signaling pathway plays an important role in maintaining immune tolerance. Blocking this pathway by sintilimab may break immune tolerance, leading to the production of autoantibodies (such as anti-GFAP antibodies) and autoimmune responses against the CNS (29). Third, the role of CD8 T cells: Previous studies have shown that Autoimmune GFAP Astrocytopathy is characterized by increased CD8 T cells in the brain tissue, which can infiltrate into the white matter and gray matter through small blood vessels, causing damage to astrocytes and triggering neuroinflammation (30). In this case, the patient had no prior viral infection or autoimmune disorders, and neurological symptoms emerged shortly after sintilimab infusion. While this tight temporal relationship strongly implies sintilimab-related immune dysregulation as a likely trigger, we acknowledge that definitive causal proof cannot be established based solely on this single case.

In this case, high-throughput microbial nucleic acid sequencing of CSF identified only trace latent Epstein-Barr virus (EBV) nucleic acid fragments, without any evidence of active EBV infection. EBV is a well-documented trigger of central nervous system autoimmunity via molecular mimicry, latent immune activation and persistent inflammatory stimulation. Combined with sintilimab-mediated immune tolerance breakdown, latent EBV reactivation in the central nervous system may synergistically amplify anti-GFAP autoimmune responses. However, we cannot distinguish whether CSF EBV represented incidental latent reactivation, transient peripheral viral translocation into CSF, or a primary pathogenic driver of astrocytopathy. During hospitalization, the patient lacked hallmark clinical manifestations of primary acute EBV encephalitis, such as persistent high fever, severe meningeal irritation, or EBV-associated lymphoproliferative lesions; therefore, targeted antiviral therapy was not initiated. Notably, the detection of trace latent EBV fragments in CSF seemingly conflicts with the exclusion of viral encephalitis; this apparent contradiction can be resolved by distinguishing asymptomatic low-level latent viral reactivation from clinically significant lytic EBV infection capable of inducing CNS inflammation. On this basis, active viral CNS infection was reliably excluded. We speculate latent EBV reactivation may synergistically amplify CNS autoimmunity associated with ICI exposure rather than acting as the primary pathogenic driver.

We further analyzed the potential correlation between the patient’s prior resolved HBV infection and the onset of immune-associated GFAP astrocytopathy. Chronic viral hepatitis can remodel systemic immune homeostasis and create a predisposition to autoimmunity via sustained low-grade immune stimulation. However, this patient exhibited complete serological resolution of HBV, negative peripheral HBV-DNA, and no active liver inflammation before sintilimab administration. No HBV nucleic acid fragments were captured in CSF mNGS testing, which excludes central nervous system HBV reactivation as a direct trigger. Collectively, prior recovered HBV infection cannot be regarded as a primary pathogenic factor for this GFAP astrocytopathy. Nevertheless, historical viral immune priming may synergistically amplify central nervous system immune dysregulation associated with PD-1 blockade, which cannot be fully excluded as a minor predisposing factor.

The treatment of autoimmune GFAP astrocytopathy associated with PD-1 inhibitor therapy mainly includes permanent discontinuation of the immune checkpoint inhibitor suspected to be linked to neurological adverse events, immunosuppressive therapy, and symptomatic supportive treatment. Discontinuation of the PD-1 inhibitor is the first step, and restarting the ICI is generally not recommended unless the potential benefits outweigh the risks. Immunosuppressive therapy is the core of treatment, with high-dose glucocorticoid pulse therapy as the first-line treatment. Glucocorticoids can effectively inhibit neuroinflammation, reduce astrocyte damage, and relieve clinical symptoms. For patients who do not respond well to glucocorticoids alone, IVIG or plasma exchange can be added. In some severe cases, immunosuppressants such as mycophenolate mofetil or rituximab may be used for maintenance treatment. In this case, a treatment regimen was employed consisting of 5-day cycles of 1000 mg methylprednisolone pulse injections combined with intravenous immunoglobulin (IVIG) at a total dose of 2 g/kg, followed by a planned 6-month gradual oral prednisone tapering protocol; oral steroid maintenance was temporarily suspended after 3 months of stable remission under joint clinical evaluation. Serial follow-up revealed negative cerebrospinal fluid GFAP antibody levels and normalization of neuroimaging parameters, confirming sustained clinical remission. In this ESCC case, single-agent paclitaxel chemotherapy was adopted as an alternative anti-tumor regimen after sintilimab withdrawal, balancing anti-tumor efficacy and the risk of recurrent neuroautoimmunity. Re-challenging with any PD-1/PD-L1 inhibitor was abandoned given the severe GFAP astrocytopathy episode, and radiotherapy was temporarily deferred to avoid superimposed systemic immune activation that might re-trigger central neuroinflammation. Regular radiological surveillance of esophageal tumor lesions was scheduled to dynamically monitor tumor progression during chemotherapy maintenance.

Importantly, all immunosuppressive regimens described above for ICI-related autoimmune GFAP astrocytopathy are applied empirically; no large-scale prospective clinical trials have validated standardized treatment protocols for this specific neurological irAE to date. Most patients with autoimmune GFAP astrocytopathy respond well to immunosuppressive therapy, with significant improvement in clinical symptoms and imaging abnormalities within weeks. However, approximately 20%-30% of patients may experience relapse, particularly those with high antibody titers or delayed treatment (31). The prognosis of autoimmune GFAP astrocytopathy is associated with primary disease control, timing of treatment initiation, and presence of comorbidities. Early diagnosis and prompt immunosuppressive therapy can lead to significant relief of neurological symptoms in most patients. In this case, the patient received treatment immediately after symptom onset, employing a high-dose glucocorticoid pulse therapy combined with IVIG, which ultimately achieved complete remission with a favorable prognosis confirmed by 3 months of continuous follow-up as of manuscript revision.

This demonstrates that early identification and timely intervention are critical for improving outcomes in patients with sintilimab-associated autoimmune GFAP astrocytopathy.

## Data Availability

The original contributions presented in the study are included in the article/supplementary material. Further inquiries can be directed to the corresponding authors.
